# Donor-derived stem-cells and epithelial mesenchymal transition in squamous cell carcinoma in transplant recipients

**DOI:** 10.18632/oncotarget.6359

**Published:** 2015-11-22

**Authors:** Laurence Verneuil, Christophe Leboeuf, Guilhem Bousquet, Charlotte Brugiere, Morad Elbouchtaoui, Louis-François Plassa, Marie-Noelle Peraldi, Celeste Lebbé, Philippe Ratajczak, Anne Janin

**Affiliations:** ^1^ INSERM, UMR_S1165, Paris, F-75010, France; ^2^ Department of Pathology, Université Paris Diderot, UMR_S1165, F-75010 Paris, France; ^3^ Department of Dermatology, CHU Caen, Caen, F-14033, France; ^4^ Université de Caen Normandie, Medical School, Caen, F-14000, France; ^5^ Department of Pathology, AP-HP, Hôpital Saint-Louis, Paris, F-75010, France; ^6^ Department of Nephrology, AP-HP, Hôpital Saint-Louis, Paris, F-75010, France; ^7^ Department of Dermatology, AP-HP, Hôpital Saint-Louis, Paris, F-75010, France

**Keywords:** squamous cell carcinoma, stem-cell, epithelial mesenchymal transition, kidney transplant, chimerism, Pathology Section

## Abstract

**Background:**

Skin squamous-cell-carcinoma (SCC), is the main complication in long-term kidney-transplant recipients, and it can include donor-derived cells. Preclinical models demonstrated the involvement of epithelial mesenchymal transition (EMT) in the progression of skin SCC, and the role of Snail, an EMT transcription factor, in cancer stem-cell survival and expansion.

Here, we studied stem-cells and EMT expression in SCCs and concomitant actinic keratoses (AK) in kidney-transplant recipients.

**Methods:**

In SCC and AK in 3 female recipients of male kidney-transplants, donor-derived Y chromosome in epidermal stem cells was assessed using combined XY-FISH/CD133 immunostaining, and digital-droplet-PCR on laser-microdissected CD133 expressing epidermal cells.

For EMT study, double immunostainings of CD133 with vimentin or snail and slug, electron microscopy and immunostainings of keratinocytes junctions were performed. Digital droplet PCR was used to check CDH1 (E-cadherin) expression level in laser-microdissected cells co-expressing CD133 and vimentin or snail and slug.

The numbers of Y-chromosome were assessed using digital droplet PCR in laser-microdissected cells co-expressing CD133 and vimentin, or snail and slug, and in CD133 positive cells not expressing any EMT maker.

**Results:**

We identified donor-derived stem-cells in basal layers and invasive areas in all skin SCCs and in concomitant AKs, but not in surrounding normal skin.

The donor-derived stem-cells expressed the EMT markers, vimentin, snail and slug in SCCs but not in AKs. The expression of the EMT transcription factor, SNAI1, was higher in stem-cells when they expressed vimentin. They were located in invasive areas of SCCs. In these areas, the expressions of claudin-1 and desmoglein 1 were reduced or absent, and within the basal layer there were features of basal membrane disappearance.

Donor-derived stem cells were in larger numbers in stem cells co-expressing vimentin or snail and slug than in stem cells not expressing any EMT marker.

**Conclusion:**

We identified here donor-derived stem cells within skin SCC in kidney-transplant recipients. They were located in invasive areas of SCC and had EMT characteristics.

## INTRODUCTION

Cancer, particularly skin squamous-cell carcinoma (SCC), is the main complication in long-term transplanted patients [1]. Preclinical models demonstrated the involvement of epithelial mesenchymal transition (EMT) in the progression of skin SCC [2], and the role of Snail, an EMT transcription factor, in cancer stem-cell survival and expansion [3].

In kidney-transplant recipients, we recently identified donor-derived epithelial cells in skin SCC and actinic keratosis (AK) [4], but the type of donor cell that homed to the skin remained to be characterized.

Here, we focused on donor-derived cells in skin SCC and AK to address the questions of stem-cell identification and EMT marker expression.

## RESULTS

### Donor-derived cells in SCC and AK expressed CD133

XY-FISH analyses performed in four female recipients of male kidney transplants, without earlier male pregnancies, showed keratinocytes with donor genotype in SCC and AK in Patients 1,2, and 3 (Table 1), and recipient genotype in Patient 4.

**Table 1:** XY-FISH data in AK and SCC in three female recipients of male kidney transplants

Sex		Kidney transplantation		Skin tumors		XY-FISH analyses		
Recipient / Donor		Age attransplant (years)	Time lapse transplant-tumor (years)	Site	Type	Keratinocytes from basal layers and invasive areas		
						XX cells %	XY cells %	% Chimeric cells corrected*
**Skin tumors in female recipients of male kidney-transplants**								XY cells
1	F/M	43	12	leg	SCC	53.5	5.3	8.3
			12	cheek	AK	65.7	2.0	2.7
2	F/M	46	5	arm	SCC	61.7	3.9	6.2
			5	neck	AK	65.3	2.4	3.3
3	F/M	18	6	arm	SCC	54.9	5.9	9.3
			6	hand	AK	67.0	1.1	1.5
								mean XY cells for SCC = 7.9
								mean XY cells for AK = 2.5
								**p value SCC vs AK = 0.05**
**Skin tumors of a male recipient of a male kidney-transplant**								
	M/M	53	6	nose	SCC	0.0	54.5	
			6	cheek	AK	0.0	58.7	
**Skin tumors of male patients without kidney transplantation (control)**								
	M			nose	SCC	0.0	66.1	
				cheek	AK	0.0	76.0	
	M			leg	SCC	0.0	60.6	
				leg	AK	0.0	70.1	

* Tumor samples from two males with SCC and AK without kidney transplantation (controls) were examined. To determine the efficiency of sex-chromosome detection in basal layers of SCC and AK, a FISH XY protocol was applied. Here the normalization factor was 1.37 and 1.58 for XX and XY cell detections of AK and SCC respectively. (p<0.05, Khi-square test)

**Figure 1:**
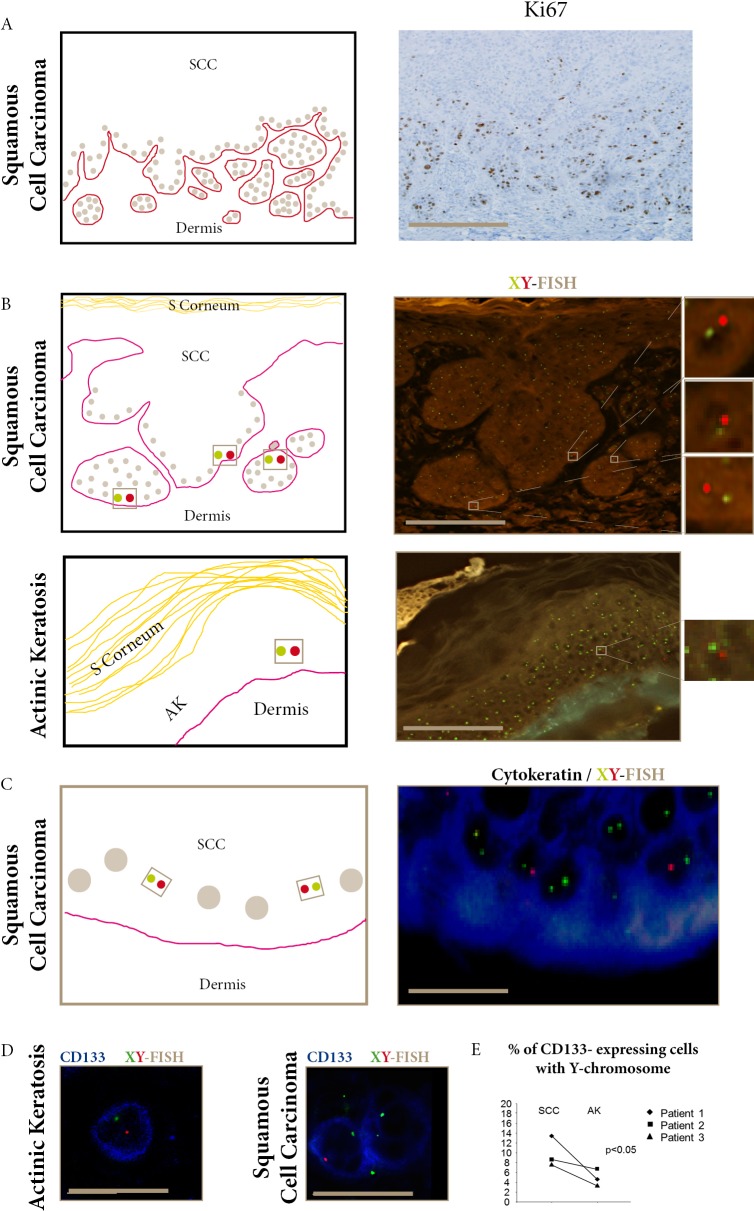
Donor- derived cells in SCC and AK express CD133 **A.** Ki67 staining of proliferative epidermal cells in the basal layer and invasive areas of SCC (bar=100μm). **B.** FISH-X (green) Y (red) show donor-derived XY cells in SCC invasive areas and AK epidermal basal layers (bars=100μm). **C.** Combined FISH-X (green) Y (red) and AE1/AE3 (blue) show donor-derived XY in cytokeratin-expressing cells in SCC basal layer (bar=15μm). **D.** Combined FISH-X (green) Y (red) and CD133 (blue) show donor-derived XY in CD133 expressing cells in AK and SCC (bar=15μm). **E.** The percentage of CD133-expressing cells with the Y-chromosome detected using droplet digital PCR is significantly higher in SCC than in AK in the three female recipients of male kidney-transplants. (p<0.05, Khi-square test).

Donor-derived XY cells (Figure 1) were distributed in the epidermal basal layers and invasive areas in the three SCC, and in the epidermal basal layers in the three AK. In 100 keratinocytes counted in each sample, the mean percentage of donor-derived XY cells was significantly higher in the three SCC (7.9%) than in the three AK (2.5%), (p<0.05) (Table 1). No donor-derived cell was found in non-tumoral tissue surrounding the SCCs.

When we assessed the expression of CD133, a marker for stem-cells, we found that CD133-expressing cells were also distributed in the epidermal basal layers in AK and in the proliferating outer cell layers in SCC. In 100 keratinocytes counted in each sample, we found a significantly higher mean percentage of CD133-expressing cells in SCC (1.8%) than in AK (0.3%) (p<0.05).

To further study if some CD133-expressing cells were donor-derived, we used i) combined CD133 and XY-FISH (Figure 1D), and ii) laser-microdissection of CD133-expressing cells (Supplementary Figure 1) followed by Y-chromosome detection using droplet-digital-PCR (ddPCR). These two independent methods identified donor-derived CD133-expressing cells in SCC and AK. ddPCR quantification showed that the mean percentage of Y-chromosome in CD133 laser-microdissected cells was significantly higher in SCC (9.8%) than in AK (4.8%) (p<0.05).

Taken together, these results demonstrated that donor-derived CD133-expressing stem-cells were present in SCCs and AKs in three kidney-transplant recipients.

### Donor-derived stem-cells expressing EMT markers were found in SCCs but not in AK

The presence of donor-derived CD133 expressing cells in invasive areas of SCC led us to study if they expressed markers of proliferation and/or invasion.

**Figure 2:**
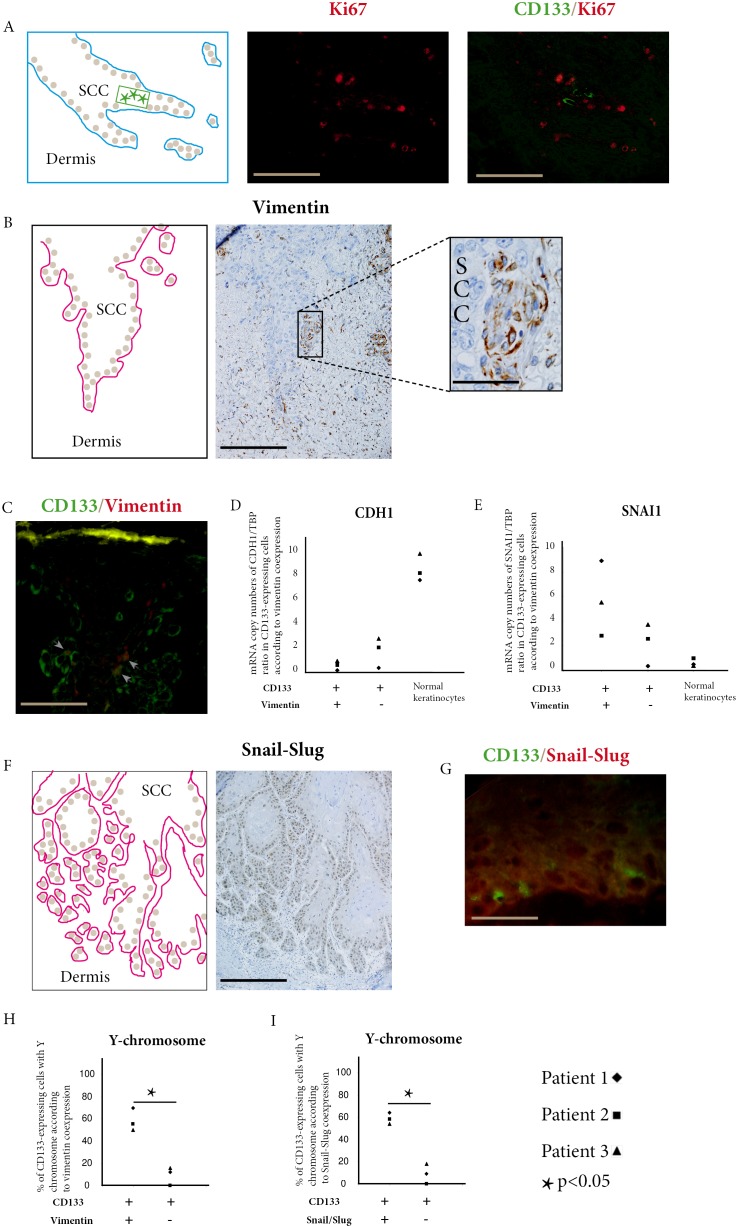
Donor-derived stem cells express EMT markers in squamous cell carcinoma **A.** Combined CD133 (green) and Ki67 (red) immunofluorescent stainings show CD133 expressing cells in SCC invasive areas. These cells do not co-express Ki67 (bar=100μm). **B.** Immunoperoxydase staining of vimentin within cells of SCC basal layer (bar=100μm, higher magnification bar=25μm). **C.** Combined CD133 (green) and vimentin (red) immunofluorescence stainings show double positive cells (arrow heads) in SCC outer cell layers (bar=25μm). **D.** In SCCs of the three kidney-transplant recipients studied, CDH1 (E-cadherin) is under-expressed in cells co-expressing CD133 and vimentin compared with cells only expressing CD133, and with normal keratinocytes. **E.** In the same patients, SNAI1 (Snail1) is overexpressed in cells co-expressing CD133 and vimentin compared with cells only expressing CD133, and with normal keratinocytes. **F.** Immunoperoxydase staining of Snail-Slug within cells of SCC basal layer and invasive areas (bar= 150μm). **G.** Combined CD133 (green) and Snail-Slug (red) immunofluorescent stainings show CD133 expressing cells in SCC outer cell layers. (bar=25μm). **H.** In SCCs of the three kidney-transplant recipients studied, cells co-expressing CD133 and vimentin have more Y-chromosome detected by droplet digital PCR than cells only expressing CD133 (p<0.05, Chi-square test). **I.** In SCCs of the three kidney-transplant recipients studied, cells co-expressing CD133 and Snail-Slug have more Y-chromosome detected by droplet digital PCR than cells only expressing CD133 (p<0.05, Chi-square test).

Double immunofluorescence of CD133 and Ki67 showed that CD133 expressing were located in invasive areas of SCC with numerous Ki67 expressing cells (Figure 2A), but we did not find cells co-expressing CD133 and Ki67 in these areas.

Since EMT is an important driver in cancer invasion, we studied the expression of vimentin and snail/slug in invasive areas of SCC.

Vimentin expressing cells were found in invasive areas of SCC (Figure 2B) and some of these cells co-expressed CD133 (Figure 2C). Counts performed in these areas showed that 11.4%, 8.9%, and 6.8% of CD133 cells co-expressed vimentin in Patients 1, 2, and 3 respectively (mean=9.0%). When we laser-microdissected cells only expressing CD133, and cells co-expressing CD133 and vimentin, we found a lower expression of CDH1 (E-cadherin) (Figure 2D) and a higher expression of SNAI1 (Figure 2E) in cells co-expressing CD133 and vimentin than in cells only expressing CD133.

In the three AK studied, no cell co-expressing CD133 and vimentin was found (Supplementary Figure 2).

Snail/slug expressing cells were numerous in basal layer and invasive areas of SCC (Figure 2F) and some of them co-expressed CD133 (Figure 2G). Counts performed in these areas showed that 10.4%, 7.5%, and 5.6% of CD133 cells co-expressed snail/slug in Patients 1, 2, and 3 respectively (mean=7,8%).

To study if CD133/vimentin or CD133/snail-slug co-expressing cells were donor-derived in the three SCCs studied, we laser-microdissected these cells and used ddPCR to detect the Y-chromosome. The percentage of Y-chromosome-bearing cells in CD133/vimentin coexpressing cells was 69%, 54.5%, and 49.5% for Patients 1, 2, and 3 respectively (mean 57. 7%). These percentages were significantly different from the percentages of Y-chromosome-bearing cells in cells expressing CD133 but not vimentin in basal layer and invasive areas of SCC (12%, 0%, and 16% for Patients 1, 2, and 3 respectively, mean 9.3%) (Figure 2H).

Similarly the percentages of Y-chromosome-bearing cells in CD133/snail-slug co-expressing cells (65.5%, 59%, and 54% for Patients 1, 2, and 3 respectively, mean 59.5%) was significantly higher than in cells expressing CD133 but not snail/slug in basal layers and invasive areas of the SCCs, (10.5%, 0%, and 18.5% for Patients 1, 2, and 3 respectively, mean 9.7%) (Figure 2I).

**Figure 3:**
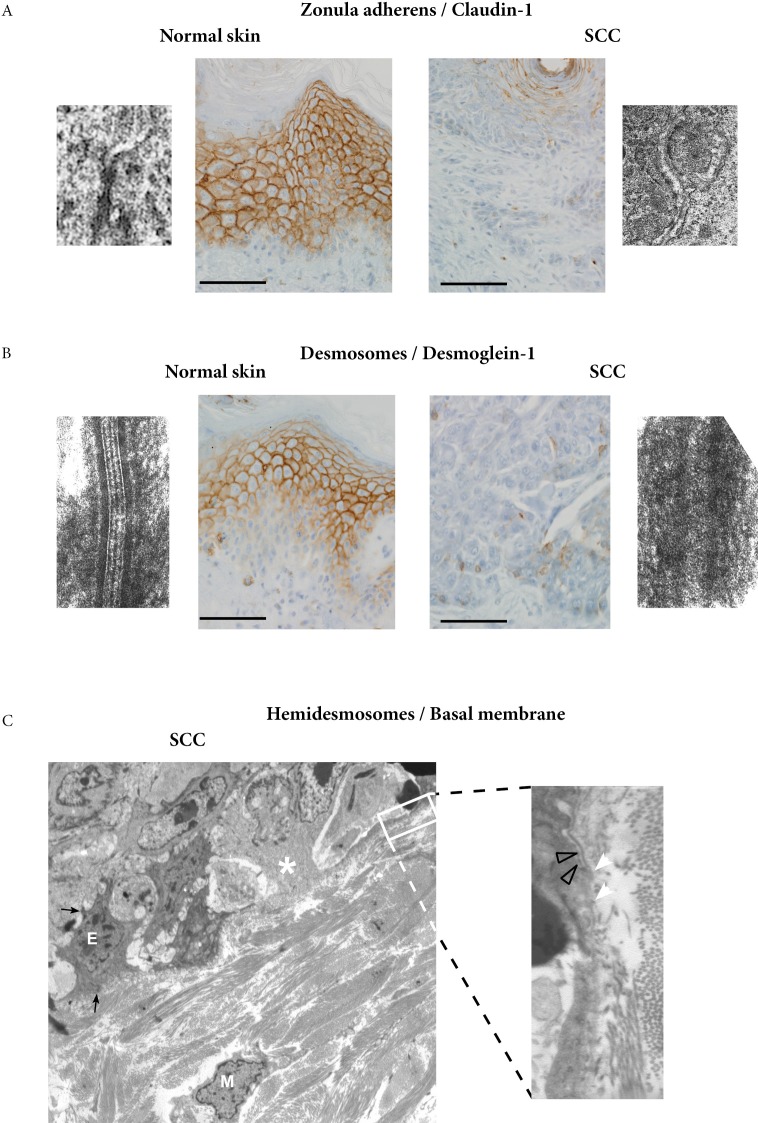
Ultrastructural study of invasive areas of SCC **A.** Comparison of normal skin and invasive area of SCC shows a partial disappearance of zonula adherens, a fact confirmed by immunoperoxydase staining of claudin-1 (bar=25μm). **B.** Comparison of normal skin and invasive area of SCC shows a partial disappearance of desmosomes, a fact confirmed by immunoperoxydase staining of desmoglein-1 (bar=25μm). **C.** Epithelial cells (E) with remaining desmosomes (black arrows) and mesenchymal cells (M) in the dermis, are close to cells within the basal layer (white asterisk) with scarce junctions and basal membrane disappearance, possibly indicative of a lessening of the epithelial characteristics (X 4000). Higher magnification shows basal membrane disappearance (white arrow heads) which can be compared with preserved basal membrane areas (grey arrows heads) (X30000).

Ultrastructural study focused on invasive areas of SCC. The systematic analysis of basal keratinocyte junctions showed a reduced number of zonula adherens and desmosomes, with partial disappearance of their dense components. On paraffin sections, immunoperoxydase staining of claudin-1 also showed a diminution of zonula adherens (Figure 3A), and immunostaining of desmoglein-1 showed a diminution of desmosomes (Figure 3B) in the same areas. For hemi-desmosomes and basal membrane, we found cells within the basal layer with scarce junctions and basal membrane disappearance, features in favour of a lessening of the epithelial characteristics (Figure 3C).

## DISCUSSION

In skin SCC from kidney-transplant recipients, we identified donor-derived stem-cells in basal layer and invasive areas. Combined laser-microdissection and molecular analyses enabled us to show that these donor-derived stem cells expressed higher levels of EMT markers than recipient stem cells.

We previously provided evidence for a donor epithelial cell contribution to the skin SCC in kidney-transplant recipients [5]. Here, to study whether stem-cells were among the donor-derived cells that homed to the recipient skin SCC, we used the Y-chromosome as a marker for male donor cells in female recipients and CD133 as a stem-cell marker [6]. In the choice of CD133 as a stem cell marker, we took into account: i) the fact that the donor-derived cells were issued from a kidney transplant, because kidney stem cells express CD133 [7], and ii) the fact that numerous studies characterized CD133 as a human cancer stem cell marker [8–16], particularly in human primary skin SCC [17].

Using two independent methods, we identified for the first time donor-derived stem-cells in SCC of kidney-transplant recipients.

Their presence in concomitant AK suggests that the homing of stem-cells from the kidney transplant to the skin also occurred at a preneoplastic stage.

The absence of donor-derived stem-cells in the normal skin surrounding SCCs and AKs could be linked to the tissue remodelling accompanying disease progression since stem-cells are recruited to sites of experimental skin injuries [18], and tissue repair shares common mechanisms with stem-cell renewal in carcinogenesis [19].

These donor-derived stem-cells were more numerous in SCC than in AK, and preferentially situated in the outer basal layers corresponding to the invasive areas of SCC. Therefore we addressed the question of the participation of these donor-derived stem-cells in tumor progression in recipient skin SCC.

We first tested the proliferation using Ki67, abundantly expressed in epithelial cells of SCC invasive areas. However we did not find in these areas cells co-expressing CD133 and Ki67.

This is coherent with *in vitro* studies showing that cancer stem cells are not in a proliferative state [20, 21].

We then tested if these donor-derived stem cells participated to tumor cell invasion. An important mechanism contributing to tumor cell invasion and migration is EMT [22, 23], characterized by concomitant loss of epithelial markers and acquisition of mesenchymal markers such as vimentin in tumor cells [24–26]. *In vitro,* the acquisition of vimentin increases tumor cell invasiveness [27]. EMT markers can also be co-expressed with CD133 in cancer stem-cells in metastatic epithelial cancer [28, 29]. Here we found CD133/vimentin coexpressing cells in SCC but not in AK.

To further characterize the EMT process in CD133 expressing cells in SCC, we laser-microdissected CD133 /vimentin co-expressing cells, and compared their molecular markers with those of cells only expressing CD133 in the same SCC areas. CD133/vimentin co-expressing cells had a higher level of the transcription factor SNAI1 (SNAIL1) and a lower level of CDH1 (E-cadherin), an adhesive molecule involved in keratinocyte junctions, together with claudin-1 for zonula adherens and desmoglein-1 for desmosomes [30]. Although these CD133/vimentin co- expressing cells were not numerous, a large percentage of them was found to be donor-derived. The fact that donor- derived stem-cells expressing vimentin were found in SCC but not in AK is an argument in favour of their invasive potential.

If, in this study performed in patients' skin samples, we could demonstrate the presence of donor-derived stem cells, and their expression of EMT markers, we could not perform *in vitro* and *in vivo* experiments to search for a clonal expansion of these cells. Given the limited numbers of donor-derived stem cells that we found, it is unlikely that these cells alone drove the tumor growth. Recent studies suggest that different types of cancer stem cells could participate in the same tumor [31]. The clinical situation of gender-mismatched kidney transplantation is particularly suitable to study the heterogeneity of cancer stem cells within tumors. We demonstrate here for the first time that part of cancer stem cells in recipient SCC is donor-derived. It cannot be excluded that the different types of cancer stem cells play different roles in tumor maintenance and progression.

In conclusion, the present *in situ* study, performed on human tumors, identified donor-derived stem-cells in recipient skin SCC. It also demonstrated the contribution of donor-derived stem-cells expressing EMT markers to invasive cells in recipient skin SCC.

## MATERIALS AND METHODS

### Patients and samples

From 1991 to 2012, four females with gender-mismatched kidney-transplants and no earlier male pregnancy had SCC and AK samples remaining after the diagnosis had been established, and available recipient DNA. Patient 1, a female with membranous glomerulonephritis, had received a male kidney transplant at age 43, and treatment with azathioprine, corticosteroids, tacrolimus, mycophenolate-mofetil and cyclosporine. Patient 2, a female with a urinary malformation, had received a male kidney transplant at age 46 with the same immunosuppressive drugs. Patient 3, a female with membranous glomerulonephritis had received a male kidney transplant at age 18 with the same five drugs. Patient 4, a female with mesangial sclerosis, had received a male kidney transplant at age 24 with the same immunosuppression except for tacrolimus. The two other patients, two females with an earlier male pregnancy, were excluded from the chimerism study. The study was approved by Institutional Review Board of Hôpital Saint-Louis (Paris, France) and informed consents were obtained according to the Helsinki Declaration.

All AK and SCC biopsy samples, taken for diagnostic purposes, had been formalin-fixed and paraffin-embedded, another part was glutaraldehyde-fixed and embedded in epoxy resin.

AK and SCC were concomitant but not contiguous, and histological diagnoses were independently reviewed by two pathologists (AJ, LV).

### XY-FISH analysis

It focused on the epidermal basal layers for AK, and on the epidermal basal layers and invasive areas for SCC. For XY-FISH analysis, 5μm-thick sections were HCl-treated for 15 min, proteinase K-digested for 20 min and formaldehyde-fixed. CEP X/Y-DNA probes (Vysis, Abbott-Molecular, Illinois, U.S.A) were denatured for 10 min at 90°C, hybridized (Thermobrite, Abbot-Molecular) for 16 hours at 42°C, and Vectashield-mounted (Vector Labs, Burlingam, U.S.A). Tissue sections were analysed by two pathologists (AJ, LV) on a motorized Z-axis- microscope (BX-61-Olympus, Tokyo, Japan), alternately using bright and epi-fluorescent light. Microscope images obtained through an UPlan-FI 100x/1.3NA objective were captured using Cell-F-software. For chromosomal analysis, 10 sequential Z-stack images of the same field were captured at 0.5μm intervals. X and Y signals were counted on 100 cells in each AK and each SCC, and also on 100 cells from non-tumoral surrounding tissue.

Two controls were used for FISH YX analyses: i) AK and SCC from one male patient with male kidney transplant, ii) AK and SCC from 2 male patients without kidney transplantation, to calculate a correction factor for XY-positive cells.

Combined XY-FISH and cytokeratin or CD133 immunostainings were performed on the same sections of AK and SCC using CEP X/Y DNA probes (Vysis), followed by staining with anti–human cytokeratin mouse antibody (clone AE1/AE3; Roche Diagnostics, Mannheim, Germany), and AMCA-conjugated anti–mouse IgG horse secondary antibody (Vector Laboratories, Peterborough, United Kingdom) or CD133 (clone AC133, Miltenyi Biotec Inc., Auburn, USA). Positive cells were counted on 500 cells in each AK and each SCC, and also on 500 cells from non-tumoral surrounding tissue.

### Immunostainings and laser-microdissection

On the 7 μm-thick sections of AK and SCC, CD133 immunostainings were performed using an indirect immunoperoxidase method (Discovery/Roche) using monoclonal mouse anti-human CD133 antibody (clone AC133 Miltenyi Biotec Inc., Auburn, USA) as the primary antibody. CD133/vimentin double immunofluorescence labelling was performed on AK and SCC following paraffin sections using the same anti CD133 mouse monoclonal antibody and anti-human vimentin mouse monoclonal antibody (clone V9, Dako, Glostrup, Danemark) as primary antibodies. Since these two primary antibodies were mouse antibodies of IgG1 isotype, the anti CD133 antibody was bound to Alexa Fluor® 488, and the anti-vimentin antibody to Alexa Fluor® 594, using Apex-Alexa Fluor® kits (Invitrogen, France).

On the 7μm-thick sections of SCC, Ki67, Snail/Slug, immunostainings were performed using monoclonal mouse anti-human Ki67 antibody (clone MIB-1, Dako) and rabbit anti-human Snail/Slug polyclonal antibody (Sigma-Aldrich). Double CD133/Ki67 and CD133/snail-slug immunofluorescence labelling were performed on SCC following paraffin sections using the same anti CD133 mouse monoclonal antibody and monoclonal mouse anti-human Ki67 antibody (clone MIB-1, Dako), and rabbit anti-human Snail/Slug (ab85936, Abcam, UK) as primary antibodies. Since these two primary antibodies were mouse antibodies of IgG1 isotype, the anti CD133 antibody was bound to Alexa Fluor® 488, and the anti-Ki67 and anti –snail/slug antibodies to Alexa Fluor® 594, using Apex-Alexa Fluor® kits (Invitrogen, France). For all immunostainings, the controls were absence of primary antibody and incubation with an irrelevant antibody of the same isotype. Counts of CD133-expressing cells and of cells coexpressing CD133 and vimentin and of cells co-expressing CD133 and snail/slug were performed by two pathologists at magnification x250 on a minimum of 500 keratinocytes in the epidermal basal layers for AK and in the epidermal basal layers and invasive areas for SCC. Results were expressed as the mean percentage of positive cells.

After CD133 immunostainings in the epidermal basal layers of AK and in the epidermal basal layers and invasive areas of SCCs, a minimum of 50 CD133^+^cells and 100 CD133^−^cells were microdissected. After double CD133 and vimentin immunostainings a minimum of 50 CD133^/^vimentin co-expressing cells and 50 cells only expressing CD133 were microdissected in the same areas. After double CD133 and snail/slug immunostainings, a minimum of 50 CD133^/^snail-slug co-expressing cells and 50 cells only exressing CD133 were microdissected in the epidermal basal layers and invasive areas of SCCs. A minimum of 100 normal keratinocytes were microdissected from surrounding areas of SCCs. Laser-microdissection was performed using a PALM-Microbeam/Zeiss system with a pulsed UV-A nitrogen laser (337nm) to select cell populations after immunostaining. For each cell population, two laser-microdissections were performed to extract: i) DNA using Qiagen-kit (Qiagen/Courtaboeuf/France), ii) total RNA using Qiagen-kit (Qiagen/Courtaboeuf/France) and reverse-transcription using random primers with SuperScriptTM II Reverse Transcriptase (Invitrogen).

### Droplet digital PCR assay

ddPCR enabled us to check the specificity of laser-microdissected cell populations using CD133 (Hs01009250_m1 (NM_001145847)), CD45 (Hs00365634_g1 (NM_002838.3)), and vimentin ((Hs00185584_m1 (NM_003380.3)). Human TBP assay was used as a housekeeping gene (Hs99999910_m1, NM_003194.4).

To detect the number of cells with the Y-chromosome in CD133 expressing cells, a minimum of 50 laser-microdissected cells were studied in three selected populations: CD133/vimentin or CD133/snail-slug co-expressing cells, and cells only expressing CD133. The primers and probes were designed to target the ZFY sequences (Applied Biosystems Hs05705529_cn), using RNase P as a reference gene (Applied Biosystems 4401631). It contained specific forward and reverse primers, FAM dye-labelled ZFY probes and VIC labelled RNase P probes. The percentage of Y-bearing cells was determined as the ZFY / RNase P ratio.

For CDH1 and SNAI1 gene expression analyses, digital droplet PCR assays were performed using Hs01023894_m1 (NM_004360.3) and Hs00195591_m1 (NM_005985.3) primers (Applied Biosystems).

Droplet digital PCR assays were performed in triplicate. Controls were no template, male, and female DNA. Reaction mixes were prepared using standard Taqman primer/probe chemistry with a 2 X ddPCR Mastermix (BioRad, Laboratories, CA), a 20 X primer/probe (900/250 nM), and 5μl sample DNA template in a final volume of 20 μl.

The PCR products were analysed using QuantaSoft software (BioRad Laboratories, CA). Raw data were collected on the Bio-Rad QX100 instrument.

CD133, CD45, CDH1 and SNAI1 mRNA expressions were expressed as the copy number of each gene / TBP copy number ratio.

### Ultrastructural study and epidermal junction immunostainings

For electron microscopy, skin biopsies were fixed in 2% glutaraldehyde-buffered 0.1 M. cacodylate and embedded in epoxy resin. Ultrathin sections were stained with uranyl acetate and lead citrate. Analysis, performed on a Hitachi-7650, focused on cells and basal membranes of invasive areas of SCC.

We looked for epithelial cell characteristics such as junctions (zonula adherens, desmosomes and hemidesmosomes since epithelial cells are keratinocytes in SCC) and for mesenchymal characteristics (no junction of any type, numerous filaments within the cytoplasm extruding from the cytoplasm). On basal membranes, we looked for changes such as thinning or thickening, disappearance of the lamina densa, lamina lucida, or hemidesmosomes.

Claudin-1 and desmoglein-1 immunostainings were performed using an indirect immunoperoxidase method (Discovery/Roche) on 5μm-thick sections of SCC and on normal skin using polyclonal rabbit anti-human anti-claudin-1 antibody (Sigma-Aldrich) and anti-DSG1 (Sigma-Aldrich) as the primary antibodies

## SUPPLEMENTARY MATERIAL FIGURES


