# Supplementary material for: Donor-derived stem-cells and epithelial mesenchymal transition in squamous cell carcinoma in transplant recipients
Source: Oncotarget. 2015 Nov 22;6(39):41497–507. doi: 10.18632/oncotarget.6359 (PMC4747169; doi:10.18632/oncotarget.6359)
Supplement: Supplementary file 1 [file oncotarget-06-41497-s001.pdf]

Donor-derived stem-cells and epithelial mesenchymal transition in squamous cell carcinoma in transplant recipients

Supplementary Material

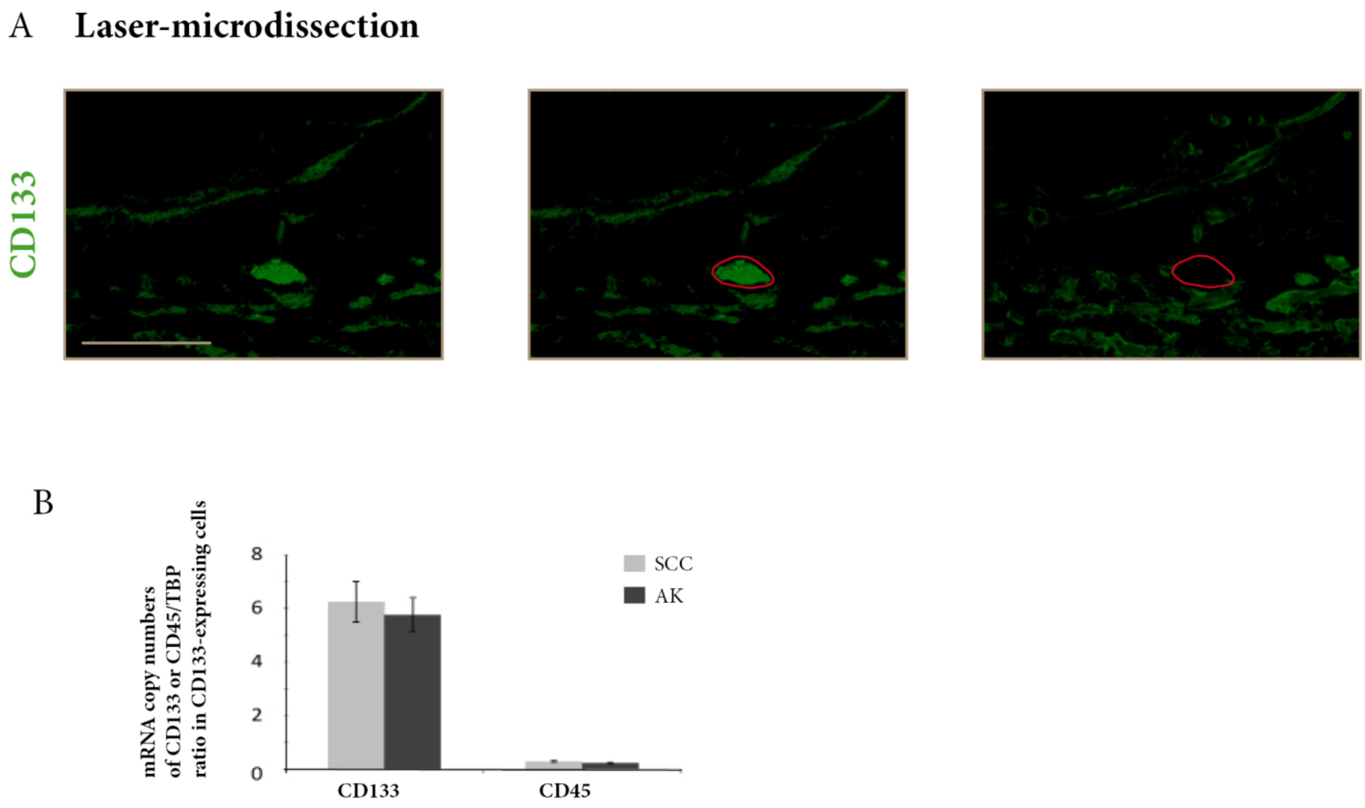

supplementary figure 1: Laser microdissection of a CD133-expressing epidermal cell (bar=30µm). Droplet digital PCR analyses show that laser-microdissected cells express CD133 (prominin 1) and not CD45

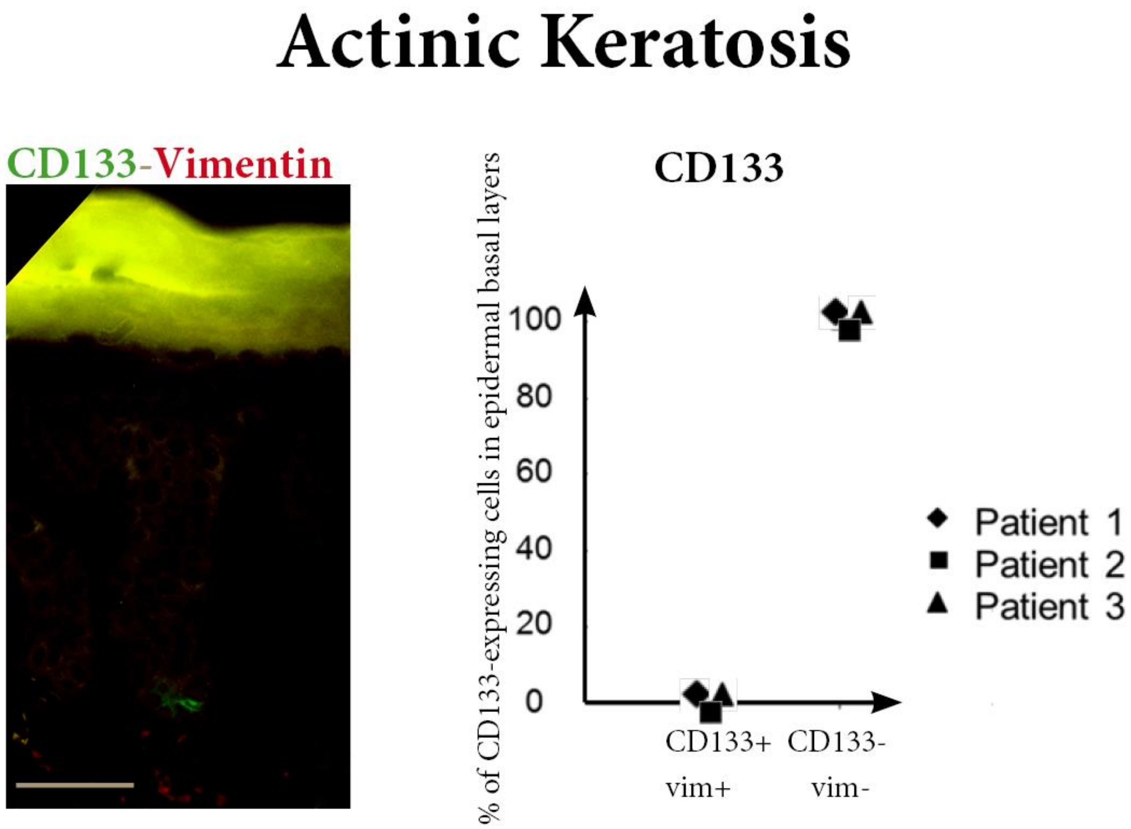

supplementary figure 2: Epidermal cells expressing CD133 but not vimentin in actinic keratoses
